# Supplementary material for: A DOPO-Based Compound Containing Aminophenyl Silicone Oil for Reducing Fire Hazards of Polycarbonate
Source: Materials (Basel). 2023 Feb 9;16(4):1449. doi: 10.3390/ma16041449 (PMC9963052; doi:10.3390/ma16041449)
Supplement: Supplementary file 1 [file materials-16-01449-s001.zip › materials-2108565-supplementary.pdf]

materials MDPI

Supplementary Materials

A DOPO-based compound containing aminophenyl silicone oil for reducing fire hazards of polycarbonate

Xiaoqing Song, Wendi Xu, Boyu Cai, Luze Wang, Zhonglin Luo and Biaobing Wang *

Jiangsu Key Laboratory of Environmentally Friendly Polymeric Materials, School of Materials Science and Engineering, Jiangsu Collaborative Innovation Center of Photovoltaic Science and Engineering, Changzhou University, Changzhou, Jiangsu, 213164, China
* Correspondence: biaobing@cczu.edu.cn; Tel.: +86 0519-8633-0075

Experimental

Materials

Polycarbonate (PC, LG 1300-03) was purchased from Korea LG Co., Ltd. (Seoul, Korea), DOPO was acquired from Aladdin Reagents Co., Ltd. (Shanghai, China). Aminophenyl silicone oil (APSO, SIC 6420, 0.3% nitrogen content) was offered from Guangzhou Silicon Carbon New Material Co., Ltd. (Guangzhou, China). Methylene chloride (CH_2Cl_2) and carbon tetrachloride (CCl_4) were purchased from Shanghai Lingfeng Chemical Reagent Co., Ltd. (Shanghai, China). Triethylamine was supplied by Jiangsu Qiangsheng Functional Chemical Co., Ltd. (Nantong, China). All chemicals were used without further purification.

Physical Characterization

TGA-FTIR was performed under a nitrogen atmosphere from room temperature to 800 °C with about 20 mg of sample and nitrogen flow of 20 mL/min.

The Limiting oxygen index (LOI) was measured by a HC-2 oxygen index meter (Jiang Ning Co., Ltd., Nanjing, China) according to GB/T 2406-93 standard and the specimen dimension used for test was 130×6.5×3 mm^3.

The vertical burning test (UL-94) was performed on a CZF-5 instrument (Shine Ray Instrument Co., Ltd, Nanjing, China) according to GB/T 2048-2008 standard and the specimen dimension used for test was 130×13×3 mm^3.

The cone calorimeter test (CCT) was conducted using a cone calorimeter (FTT, East Grinstead, UK) according to ISO 5660-1 standard with a thermal radiation power of 35 kW/m^2, and the dimension of square sample was 100×100×3 mm^3.

Scanning electron microscopy (SEM) with energy dispersive spectroscopy (EDS) was carried out by field emission scanning electron microscope (Zeiss SUPRA55, Jena, Germany) was used to observe the morphology of the residual carbon after CCT and the microscopic morphology of the impact-fractured surface of the sample.

X-ray photoelectron spectroscopy (XPS) was determined by a ESCALAB 250XI X-ray photoelectron spectrometer (Oxford company, UK).

The tensile measurement was performed on a WDT-30 universal testing machine (Shenzhen Kaiqiangli experimental Instrument Co., Ltd., Guangzhou, China) according to GB/T 1040–2006. The Izod notched impact strength was measured by a XBL-22 cantilever beam impact testing machine (Shenzhen Kaiqiangli Experimental Instrument Co., Ltd., Guangzhou, China) according to GB/T 1843–2008.

1. Fourier Transform Infrared (FTIR)

Fourier transform infrared (FTIR) spectra were obtained from a Spectrum II spectrometer (Perkin Elmer, USA) after 16 scans in the wavelength range of 4000-500 cm^{-1} at 4 cm^{-1} spectrum resolution.

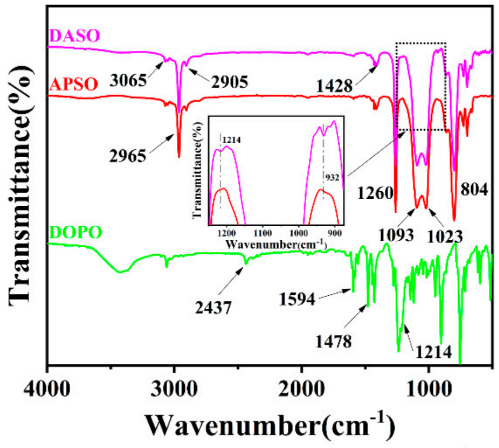

Figure S1. FTIR spectra of DOPO, APSO and DASO.

2. Nuclear Magnetic Spectrum

The proton nuclear magnetic spectrum (^{1}H NMR) and the nuclear magnetic phosphorus spectrum (^{31}P NMR) were performed on an AV II-400 MHz nuclear magnetic resonance spectrometer (Bruck, Germany) at room temperature using deuterated chloroform ($CDCl_3$) as the solvent.

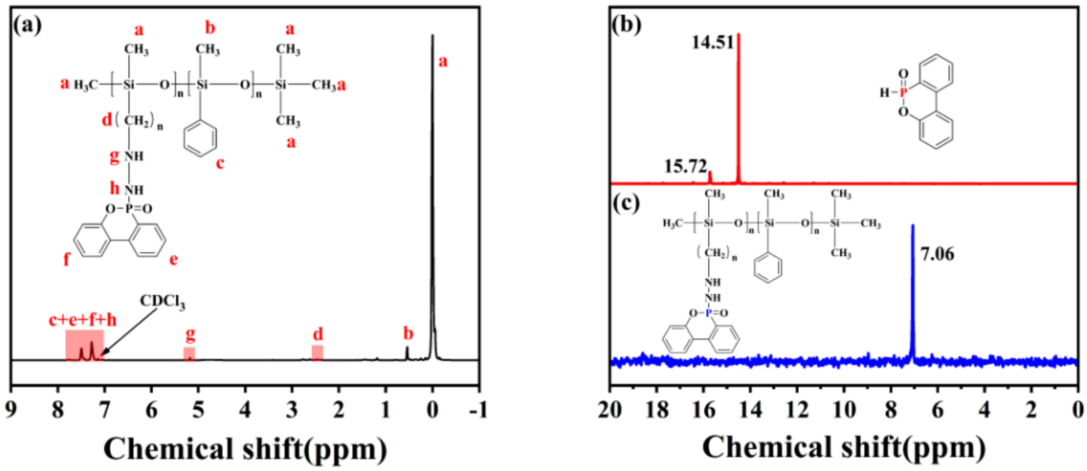

Figure S2. ^{1}H NMR spectra of DASO (a), ^{31}P NMR spectra of DOPO (b) and DASO (c).

3. Thermogravimetric Analysis (TGA)

TGA was performed on a Perkin Elmer TGA 4000 (Waltham, Massachusetts, USA) at a heating rate of 10 °C/min from room temperature to 800 °C under nitrogen and air atmosphere.

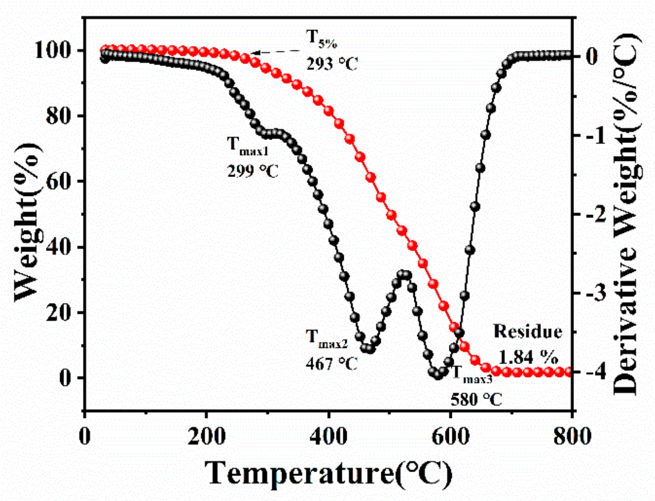

Figure S3. TGA and DTG curves of DASO.

4. Raman Spectra

Raman spectroscopy measurement was carried out on a DXR Raman spectrometer (Thermo Fischer Scientific, USA) with the excitation wavelength of 532 nm.

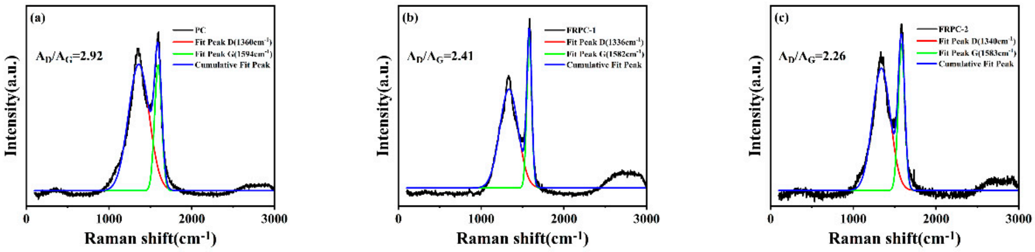

Figure S4. Raman spectra of char residues for PC (a), FRPC-1 (b), and FRPC-2 (c).

5. Absorption Intensities of Four Typical Flammable Gases with Time

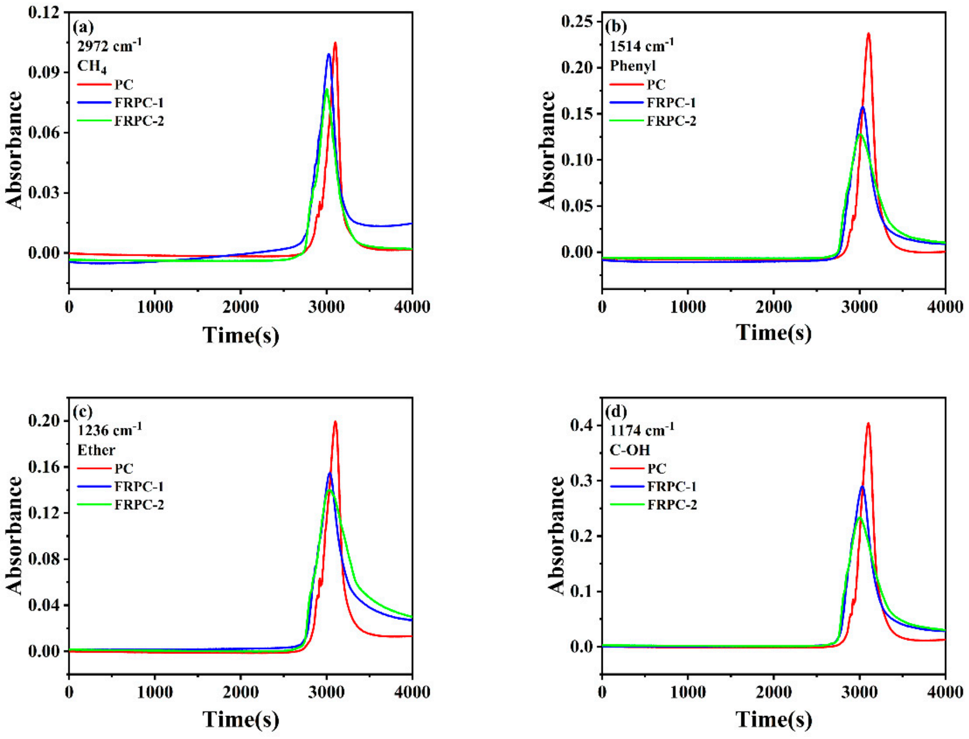

Figure S5. Absorption intensities of CH_4 (a), phenyl (b), ether (c) and C-OH (d) versus time in the thermal degradation for PC and FRPCs.
